# Thermodynamic Principles Behind Mechanisms and Reactivities: Hydrogen Atom Abstraction and Related Radical Reactions

**DOI:** 10.1021/acs.accounts.5c00879

**Published:** 2026-02-17

**Authors:** Martin Srnec, Daniel Bím, Mauricio Maldonado-Domínguez, Zuzanna Wojdyla, Radek Fučík

**Affiliations:** † J. Heyrovský Institute of Physical Chemistry, Czech Academy of Sciences, Dolejškova 3, Prague 8 18200, Czech Republic; ‡ Department of Physical Chemistry, 52735 University of Chemistry and Technology, Prague 16628, Czech Republic; § Facultad de Química, Departamento de Química Orgánica, Universidad Nacional Autónoma de México, 04510 Ciudad de México, México; ∥ Faculty of Nuclear Sciences and Physical Engineering, 156923 Czech Technical University in Prague, Trojanova 13, Prague 2 12000, Czech Republic

## Abstract

Hydrogen atom abstraction (HAA) is one of the most pervasive radical reactions in biology and chemistry. It is central to enzymatic catalysis, respiration, and photosynthesis, and underpins modern synthetic strategies like selective C–H functionalization. Despite its ubiquity, predicting HAA reactivity and selectivity remains notoriously difficult: comparably strong X–H bonds (with X = C, O, N, ...) within the same molecule often display starkly contrasting reactivities that well-known linear free-energy relationships (LFERs) often fail to capture.

In this Account, we describe how *off-diagonal* thermodynamics complements Hammond’s view of transition states as early or late as a consequence of their *diagonal* thermodynamic driving force (Δ*G*_0_). It does so by gauging the effect of proton-transfer (PT) and electron-transfer (ET) states in the *character* of a concerted HAA reaction, that is, whether the charge distribution in transition state resembles more PT or ET instead of neutral HAA. Two key descriptors are presented, asynchronicity (η) and frustration (σ), which account for the effect of PT/ET states on HAA kinetics and, together with Δ*G*_0_, they formulate a three-component thermodynamic framework. Herein, we summarize and provide a unifying view of how this framework can be utilized to provide insight and quantification of reaction outcomes, e.g., through prediction of relative barriers and selectivity, tunneling contributions, polarity effects, and even judgement of the bias in post-HAA selectivity. Extending the concept of off-diagonal thermodynamics uncovers how H atom abstraction connects to broader radical-transfer chemistries, ultimately leading to the discovery of a newly described mechanism: hydride-coupled electron transfer (HCET). By integrating thermodynamic cycles, Marcus theory, and computational analyses, we propose that off-diagonal thermodynamics provide not only a unifying language in HAA and related radical chemistry, connecting quantum chemistry and experimental measurements, but also a practical predictive tool for chemists. Looking ahead, we outline how this framework can guide experimental design, bridge the gap between adiabatic and nonadiabatic regimes, and expand beyond HAA to an extended theory of radical reactivity.

## Graphical abstract

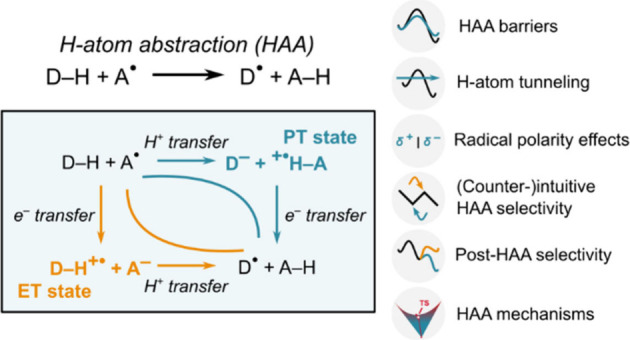

## Key References

-

Bím, D. ; Maldonado-Domínguez, M. ; Rulíšek, L. ; Srnec, M. Beyond the classical thermodynamic contributions to hydrogen atom abstraction reactivity. Proc. Natl. Acad. Sci. U. S. A. 2018, 115, E10287–E10294.30254163 10.1073/pnas.1806399115PMC6217389 ^1^ This paper introduces one off-diagonal thermodynamic factor—*asynchronicity*, which measures the imbalance of electron and proton transfer in concerted proton–electron transfer and quantifies its effect on the reaction barrier.
-

Maldonado-Domínguez, M. ; Srnec, M. H-Atom Abstraction Reactivity through the Lens of Asynchronicity and Frustration with Their Counteracting Effects on Barriers. Inorg. Chem. 2022, 61, 18811–18822.36371687 10.1021/acs.inorgchem.2c03269 ^2^ This paper introduces a second off-diagonal thermodynamic factor—*frustration*, which quantifies the penalty imposed on the reaction barrier for concerted proton–electron transfer when the H atom acceptor simultaneously exhibits increased oxidizing power and basicity, and/or when the H atom donor simultaneously exhibits increased reducing power and acidity.
-

Maldonado-Domínguez, M. ; Srnec, M. Quantifiable polarity match effect on C–H bond cleavage reactivity and its limits in reaction design. Dalton Trans. 2023, 52, 1399–1412.36644790 10.1039/d2dt04018b^3^ In this paper, we demonstrate that asynchronicity effectively represents the qualitative polarity effects on reactivity known in organic chemistry.
-

Maldonado-Domínguez, M. ; Srnec, M. Understanding and Predicting Post H-Atom Abstraction Selectivity through Reactive Mode Composition Factor Analysis. J. Am. Chem. Soc. 2020, 142, 3947–3958.32000494 10.1021/jacs.9b12800 ^4^ This paper shows that asynchronicity influences the extent of H atom motion at the transition state along the H atom abstraction trajectory, which in turn affects post-abstraction selectivity by biasing which reaction channel is favored when under dynamic control.
-

Wojdyla, Z. ; Srnec, M. Radical ligand transfer: mechanism and reactivity governed by three-component thermodynamics. Chem. Sci. 2024, 15, 8459–8471.38846394 10.1039/d4sc01507jPMC11151871 ^5^ We extend the concepts of asynchronicity and frustration to radical ligand transfer, showing that off-diagonal thermodynamics decisively governs the mechanism: among possible pathways—either electron transfer coupled to cation transfer or anion transfer—the reaction follows the thermodynamic cycle with the most favorable off-diagonal contribution to reactivity.
-

Wojdyla, Z. ; Gopinath, J. S. ; Srnec, M. Hydrogen Atom Abstraction via Hydride-Coupled Electron Transfer and its Origin. Inorg. Chem. 2025, 64, 22698–22710.41231153 10.1021/acs.inorgchem.5c03613PMC12648667 ^6^ In this paper, we report a fundamentally new mechanism for H atom abstraction. Using the example of C–H bond activation in cyclohexane by a Cu-containing complex, we identify a hydride-coupled electron transfer (HCET) mechanism, in place of conventional PCET, and attribute it to favorable off-diagonal energetics associated with the thermodynamic cycle involving electron and hydride transfers.

## Introduction

Hydrogen atom abstraction (HAA) reactions are pervasive in nature and drive a wide range of essential biological processes including, for example, enzymatic catalysis,^7^ cellular respiration,^8^ photosynthesis,^9^ carbon dioxide fixation,^10^ and nitrogen fixation.^11^ Beyond their biological significance, these reactions have also emerged as central tools in modern synthetic chemistry, enabling the construction of complex molecules. In many cases, HAA serves as the initiating step for carbon–carbon backbone bond formation, highlighting its value in the development of innovative synthetic strategies for precise molecular design (Figure 1A).^12^

**Figure 1:**
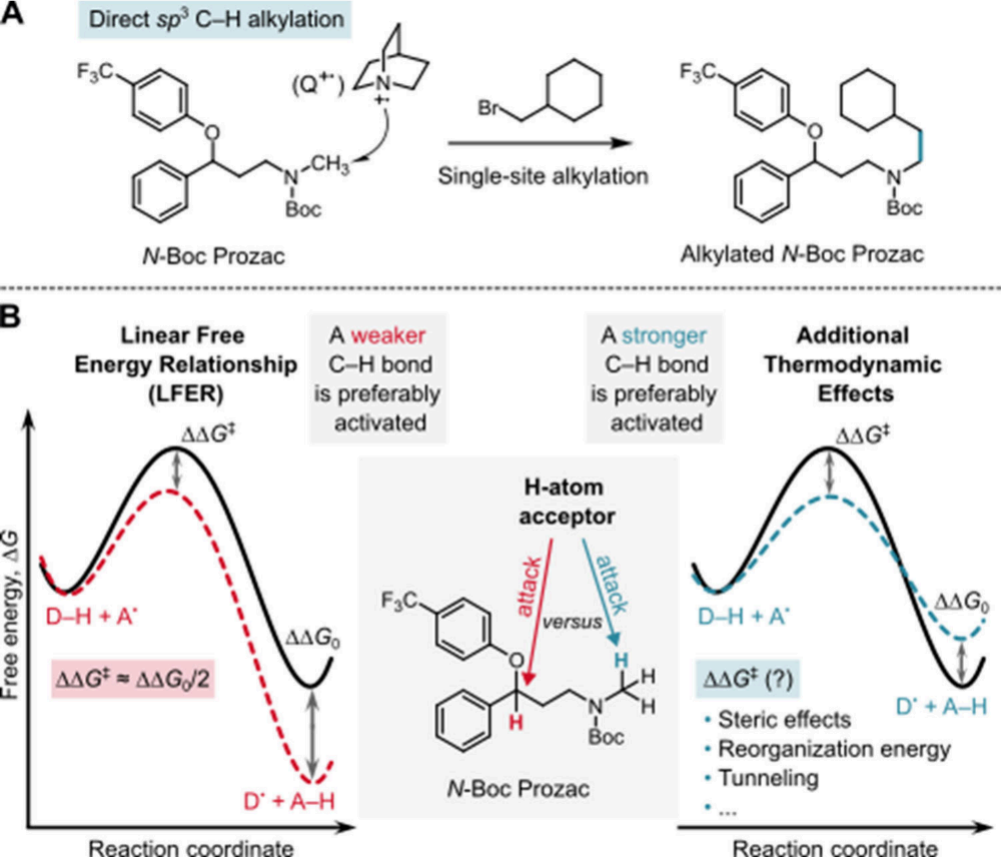
(A) Quinuclidinium radical, a key species in MacMillan’s photoredox system shows counterintuitive HAA selectivity, preferentially activating stronger C–H bonds in *N*-Boc-Prozac despite weaker ones being present.^14^ (B) Selectivity arising from barrier control. While a linear free-energy relationship (LFER) predicts greater transition-state stabilization for more stable products (left), deviations can occur (right), as in MacMillan’s study, where non-LFER behavior was attributed to a *polarity match* (see text).

To this day, accurately predicting HAA reactivity and selectivity, thereby achieving more effective control over product outcomes, remains an unresolved challenge. In pursuit of this goal, we have developed a conceptual framework centered on *off-diagonal thermodynamics*, where the fundamental and interconnected processes of HAA—electron transfer (ET) and proton transfer (PT)—serve as key predictors of reactivity and selectivity. Similar elemental processes can also be found in radical-based reactions beyond HAA. This framework forms the core of the present Account.

Before introducing off-diagonal thermodynamic terms and their connection to kinetics, it is essential to first consider a foundational and well-established principle linking classical thermodynamics (via equilibrium constant, *K*) to kinetics (via rate constant, *k*): in a series of related reactions, those with a higher *K* generally proceed more rapidly.^13^ As illustrated in the left panel of Figure 1B, this relationship corresponds to what is known in the language of Gibbs free energies as a linear free-energy relationship (LFER), in which variations in the reaction barrier (Δ*G* ^⧧^) generally track roughly half of the change in the overall reaction free energy (Δ*G*_0_). As a result, more exergonic reactions, where products are more stable relative to reactants, tend to have lower activation barriers. However, numerous exceptions to LFER exist. An example that deviates from the LFER is the HAA reaction between the quinuclidinium radical and the set of C–H bonds in Prozac.^14^ Instead of cleaving the weak benzylic C–H bond, a stronger α-amino C–H bond is preferred (Figure 1B). The original paper provides a qualitative rationale of this non-LFER selectivity through the notion of *polarity match*, which is a widely recognized factor that complements LFER and contributes to the selectivity and reactivity of radical-based systems.^15,16^ Polarity matching serves as a valuable tool for selectivity prediction and development of complex catalytic strategies.^17^

## Free-Energy Surfaces As Windows into How Thermodynamics Govern HAA Reactivity

Δ*G*_0_ represents the thermodynamic driving force for the HAA reaction, regardless of whether the reaction proceeds through a single-step pathway directly connecting reactants to products (as shown by the *diagonal* path in Figure 2A), or through two stepwise, *off-diagonal* alternatives, where (i) ET from donor to acceptor occurs first, forming an electron-transfer state, followed by PT; (ii) PT occurs first to generate a proton-transfer state, followed by ET (Figure 2A). The intuition underlying the conceptual framework of the off-diagonal thermodynamics described herein, is that the energetics of the two off-diagonal ET and PT states will indirectly influence the characteristics of the single-step (*diagonal*) HAA, even though these states are not accessed as they lie at higher energies than the transition state for the single-step HAA. Namely, these off-diagonal (ET and PT) states coshape the overall free-energy surface and, consequently, the height of the reaction barrier. This aspect is illustrated in Figure 2B, where three single-step HAA trajectories (red arrows) are depicted on three hypothetical free-energy surfaces. Let us first consider the scenarios in which the ET and PT states are energetically equivalent. Intuitively, the higher ET and PT energies (cf., left vs middle plot in Figure 2B) leads to distortion of the free-energy surface so that the single-step HAA barrier is increased (ΔΔ*G* ^⧧^ > 0). In contrast, when the ET and PT states have different energies, but maintaining the sum of their energies equal to that of the second scenario (*cf*. middle vs right plot in Figure 2B), the free-energy landscape is distorted in a manner that decreases the single-step HAA barrier (ΔΔ*G* ^⧧^ < 0).

**Figure 2:**
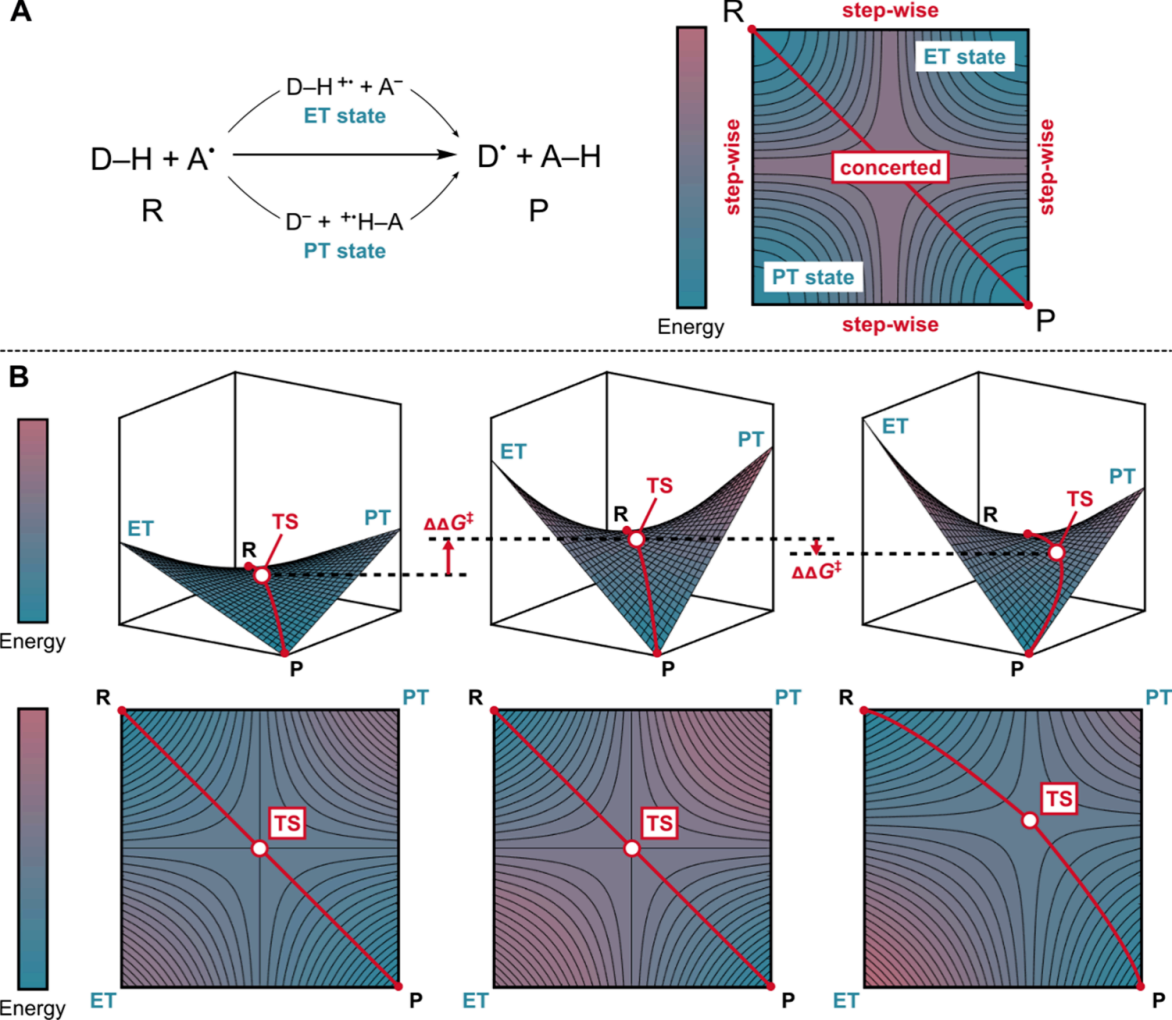
(A) Three thermodynamically equivalent pathways for HAA: a single-step HAA and two sequential (two-step) HAA mechanisms—one involving electron transfer followed by proton transfer and the other involving PT followed by ET, with corresponding intermediate ET and PT states. (B) Free energy profiles of three distinct single-step HAA reactions (depicted by red trajectories), influenced by the energetics of the ET and PT states. Adapted with permission from ref 2. Copyright 2022 American Chemical Society.

## Three-Component Thermodynamics Underlying HAA Reagents and Their Reactions

While the free-energy surface perspective offers a qualitative insight into how the ET and PT states influence single-step HAA reactivity, the crucial follow-up is to provide a quantitative description of their impact on the reaction kinetics. This requirement leads to Figure 3A, which illustrates the HAA thermodynamic cycle and its decomposition into half-reaction cycles corresponding to each of the two reactants – specifically, the H atom acceptor (A^•^) and the dehydrogenated form of the H atom donor (D^•^). These species are defined by three key thermodynamic properties: hydrogenation potential (*E*_H_^°^), one-electron reduction potential (*E*°), and acidity constant (p*K*_a_). The latter two can be further combined to yield two off-diagonal thermodynamic descriptors of the half-reactions—*potential duality* (μ) and *potential disparity* (ω)—as detailed in the equations provided in Figure 3B. The potential duality quantifies a species’ balanced strength as both an oxidant and a base, while the potential disparity reflects its relative tendency to act more effectively as an oxidant versus a base, or vice versa. Contrary to *E*_H_^°^ which describes the sequential acquisition of an electron and a proton by A^•^ (D^•^), μ expresses their simultaneous and mutually independent acquisition by A^•^ (D^•^).

**Figure 3:**
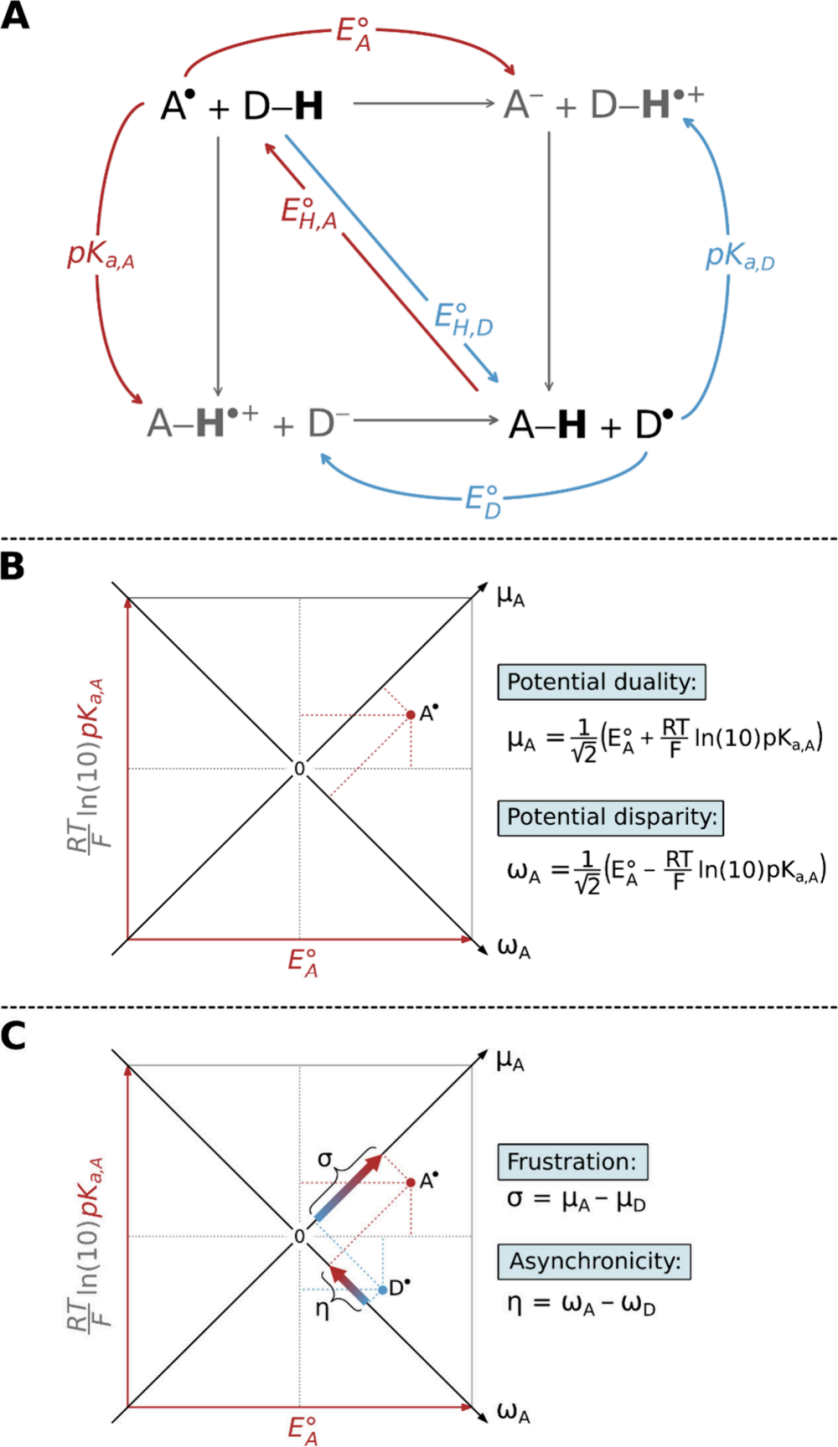
(A) Thermodynamic representation of HAA showing the full-reaction cycle and its decomposition into half-reaction cycles for the H atom acceptor A^•^ and the dehydrogenated form of H atom donor D^•^. (B) Definition of potential disparity ω and potential duality μ of A^•^, derived from experimentally and/or computationally accessible quantities p*K*_a_ and reduction potential *E*°. (C) Introduction of the off-diagonal full-reaction descriptors asynchronicity η and frustration σ that arise for a given H atom donor/acceptor pair. Adapted with permission from refs 1 and 2 . Copyrights 2018 and 2022 National Academy of Sciences and American Chemical Society, respectively.

To move from the half-reaction off-diagonal thermodynamic quantities to those describing the full reaction, we take the difference in potential dualities of the two species, A^•^ and D^•^, which defines the *frustration (σ)*, and the difference in their potential disparities, which defines the *asynchronicity (η)*—as again detailed in Figure 3C. Frustration and asynchronicity together define the basis of off-diagonal thermodynamics, while the reaction free energy Δ*G*_0_—given by the difference in hydrogenation potentials of A^•^ and D^•^—constitutes the diagonal thermodynamic component. Thus, the complete thermodynamic basis for understanding reactivity in the realm of concerted ET/PT reactions consists of three *independent* components, collectively referred to as *three-component thermodynamics*.^1,2^

## Asynchronicity as the First Off-Diagonal Thermodynamic Component: Quantified View of How It Shapes HAA Reactivity

To connect the three-component thermodynamics with the single-step HAA reactivity, one may take the advantage of the Marcus-type model for the reaction barrier, which offers a simple analytic formula depending on two quantities, Δ*G*_0_ and reorganization energy (λ):

1 $$\Delta G^{⧧}=\frac{\lambda }{4}{\left(1+\frac{\Delta G_{0}}{\lambda }\right)}^{2}\approx \frac{\lambda }{4}+\frac{\Delta G_{0}}{2}$$

Here, the right-most expression represents a linear approximation of the quadratic form, which is valid under the common assumption that λ ≫ |Δ*G*_0_|. From the equation, the last term corresponds to the well-known LFER. Using this linearized Marcus-type model, we computationally investigated a series of HAA reactions between the H atom donor, cyclohexadiene, and a set of tetramethylcyclam-supported (L)­Fe^IV^=O oxidants that share a common structural framework but differ in their axial ligand L, as illustrated in Figure 4.^1^

**Figure 4:**
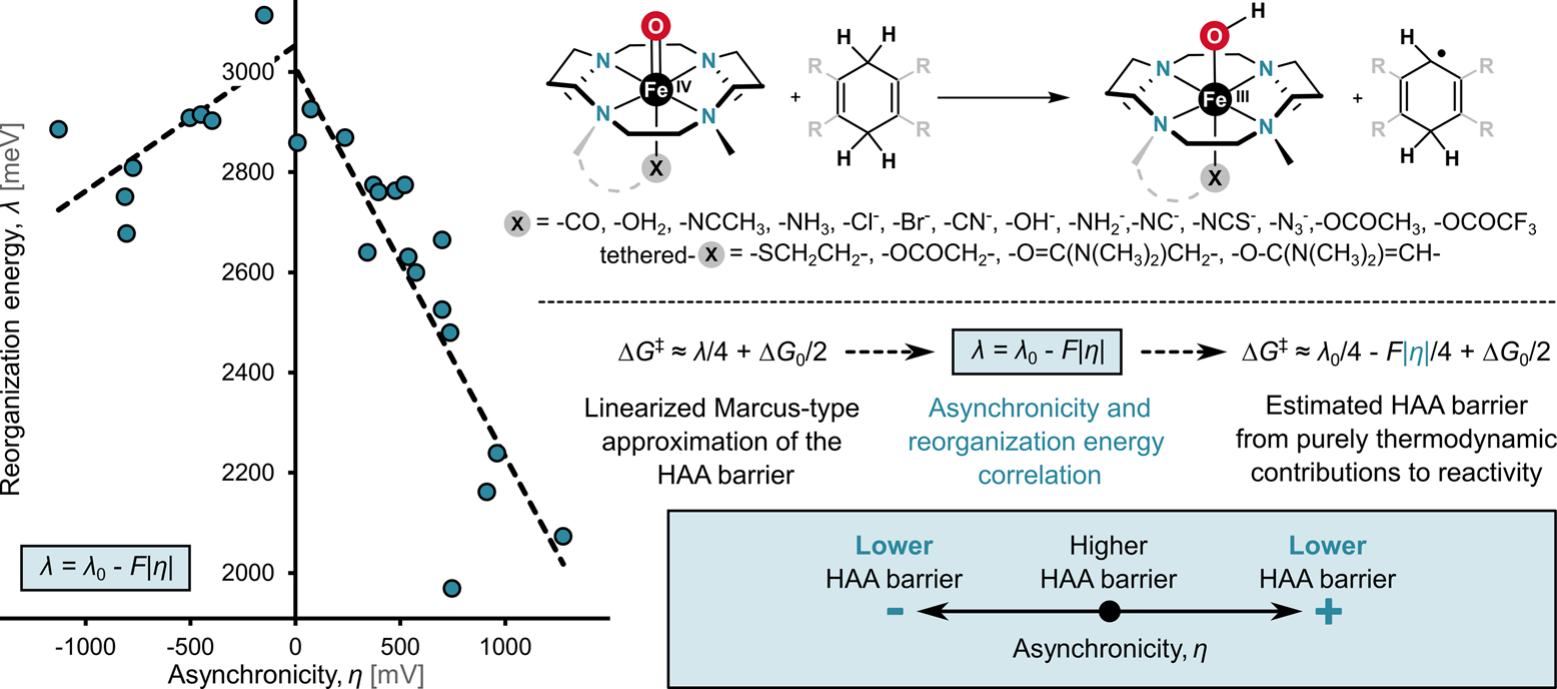
Relationship between the reorganization energy λ and the asynchronicity η for a series of HAA reactions. The plot shows a volcano-shaped dependence, with λ reaching a maximum at η ≈ 0, while increasing HAA asynchronicity (i.e., more positive or negative η) corresponds to lower λ values. Observed trend enables a modification of the classical Marcus-type expression for the HAA activation barrier and allows a direct incorporation of the thermodynamic asynchronicity factor η into predictive models for HAA kinetics. Adapted with permission from ref 1. Copyright 2018 National Academy of Sciences.

Within this series, we found a strong correlation between reorganization energy λ and asynchronicity η (Figure 4, left). This correlation exhibits two striking features: (i) changes in λ closely mirror changes in η, with an approximate 1:1 ratio and (ii) the maximum value of λ is observed when η approaches zero (denoted as λ_0_), while λ decreases regardless of whether the reaction becomes asynchronous in favor of ET (η > 0) or PT (η < 0). Incorporating the observed correlation into the right-most term of eq 1 reveals that the free-energy barrier is proportional to −|η|/4, thanks to fairly consistent frustration across the series, consistently leading to a reduction in the barrier. In this regard, it mirrors the qualitatively described effect of energy imbalance between off-diagonal ET and PT states on the single-step HAA energy profile illustrated in Figure 2B.

The term *asynchronicity* refers to a mechanistic aspect of the reaction—a disparity in the extent of ET versus PT as the system progresses from the reactant to the transition state. Indeed, as illustrated in Figure 5 for a subset of related HAA reactions with essentially the same Δ*G*_0_, asynchronicity shows a tight correlation with the charge-based descriptor Δ*q*, which quantifies the difference between the change in charge on the hydrogen atom abstractor (reflecting the extent of ET) and that on the hydrogen atom (reflecting the extent of PT) as the system evolves from reactant to transition state.^1^ As η becomes more positive—thermodynamically favoring electron transfer over proton transfer in HAA—Δ*q* increases accordingly, indicating more advanced ET relative to PT at the transition state, and vice versa. At the synchronous limit, η and Δ*q* are both equal to zero.

**Figure 5:**
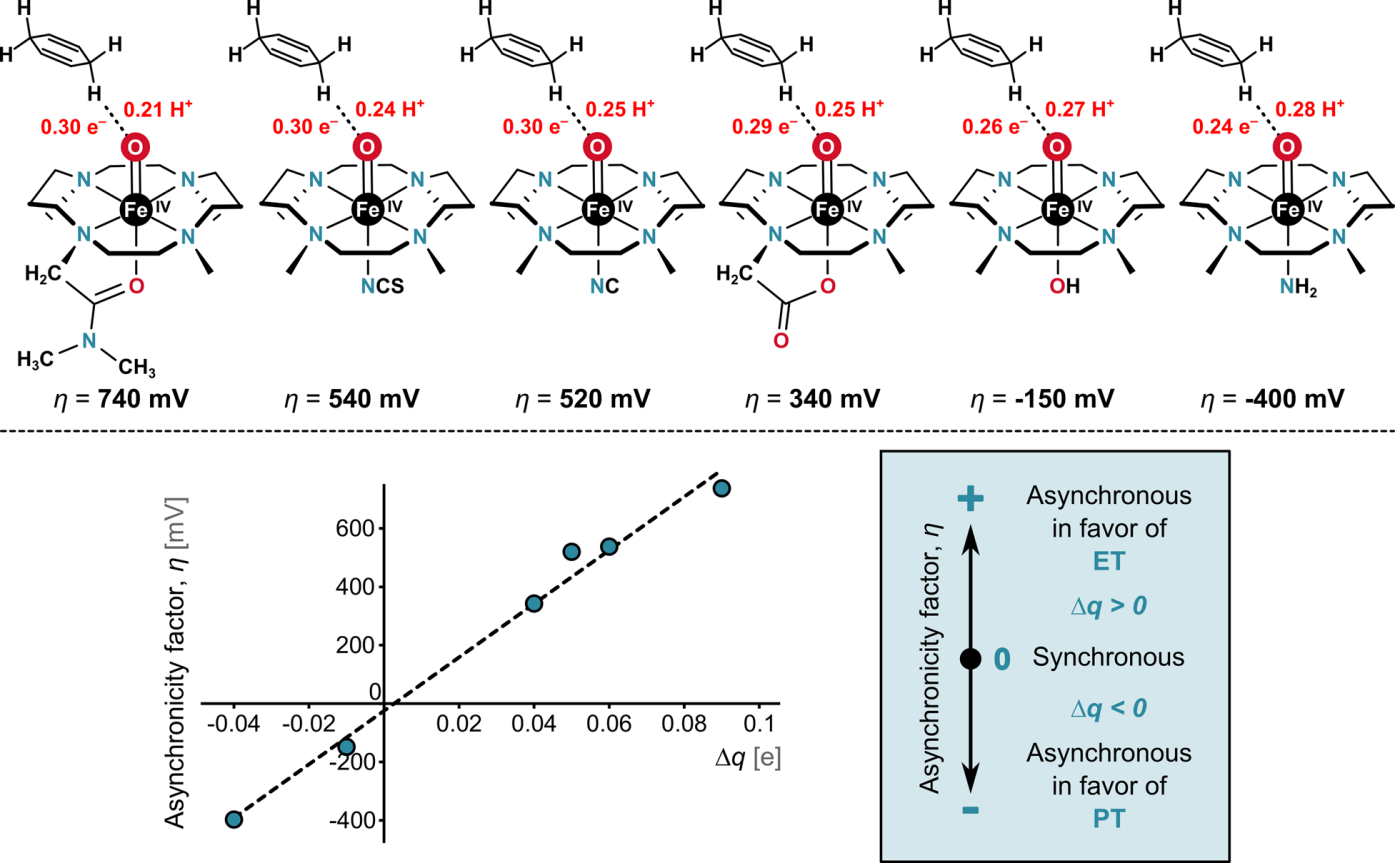
Schematic representations of transition states (TSs) for a set of HAA reactions with nearly identical reaction free energies Δ*G*_0_ but different asynchronicities η. The top panel illustrates TSs and the relative amounts of proton and electron transfer. For systems with a more positive η, the TS occurs earlier along the C–H coordinate and involves a greater degree of electron transfer (larger Δ*q*), consistent with an electron transfer (ET)-favored asynchronous mechanism. Conversely, a more negative η yields later TSs with smaller Δ*q*, consistent with a proton transfer (PT)-favored character. Adapted with permission from ref 1. Copyright 2018 National Academy of Sciences.

Of interesting note, both thermodynamically defined off-diagonal quantities and similar charge-based descriptors (for brevity denoted here as Δ*Q*; more in ref 18) correlating with asynchronicity (and with a combined effect of asynchronicity and frustration discussed later) were shown to be *additively* transferable in a space of related reactions. This means that the Δ*Q* for the reaction between reactants 1 and 2 (Δ*Q*_12_) and the Δ*Q* for the reaction between reactants 1 and 3 (Δ*Q*_13_) can be combined to obtain the Δ*Q* for the reaction between reactants 2 and 3 (Δ*Q*_23_).^18^

## Applications of Asynchronicity in Elucidating Noncanonical Reactivity/Selectivity Patterns

As of now, asynchronicity has been experimentally demonstrated and/or successfully applied to rationalize experimental findings involving metal complexes with diverse reactive units and organic radicals, as exemplified by refs 19–32 , and in our laboratory, we have also investigated and validated several other key aspects of asynchronicity, showcasing the broader chemical landscape associated with this quantity.

First, asynchronicity serves as a reliable indicator of the polarity match between the nucleophilicity or electrophilicity of the H atom abstractor and the philicity of the H atom of the donor (Figure 6), thereby allowing for the quantification of its influence on reactivity.^3^ This connection has enabled us to rationalize otherwise counterintuitive selectivities, where stronger C–H bonds are preferentially activated over weaker ones, and to provide a rigorous, quantifiable framework for the widely invoked *polarity-match effect* in H atom abstraction chemistry.

**Figure 6:**
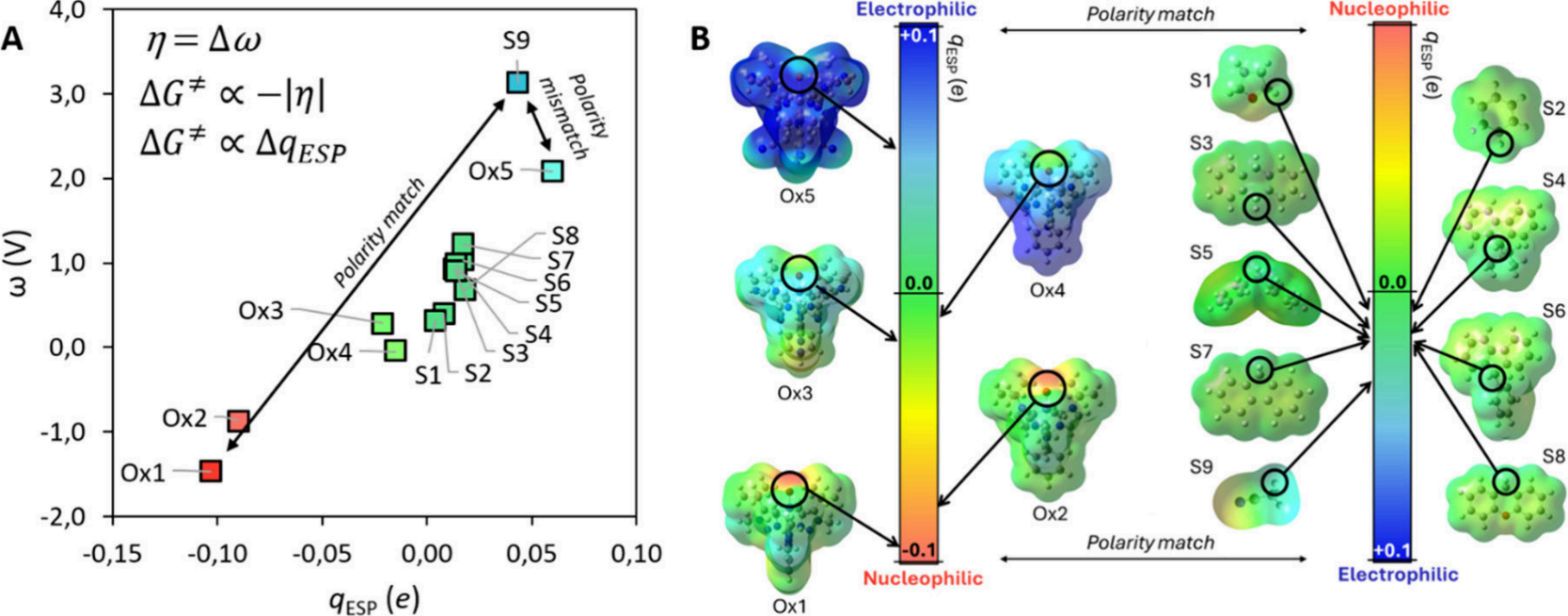
(A) Correlation between potential disparity ω and electrostatic potential charge (*q*_ESP_) for Co^III^–oxo oxidants (Ox1–Ox5) and H-donor substrates (S1–S9). The scaling of *q*_ESP_ with ω links Δ*q*_ESP_ to asynchronicity η. Substrate–oxidant pairs positioned farther apart show better polarity matching, whereas nearby pairs are mismatched. (B) ESP maps of Ox1–Ox5 and S1–S9 illustrating the shift from nucleophilic (red) to electrophilic (blue) oxygen centers. The color scale reflects *q*_ESP_ values, capturing how local polarity and thermodynamic descriptors jointly determine asynchronicity and polarity matching in HAA chemistry. Adapted with permission from ref 3. Copyright 2023 Royal Society of Chemistry.

Second, employing hundreds of organic HAA reactions, we demonstrated the existence of the *pseudoinverted region*, where the barrier increases as Δ*G*_0_ decreases (non-LFER behavior), even for moderate Δ*G*_0_ values (the proper inverted region is observed for much lower Δ*G*_0_ values and arises from quadratic effects of Δ*G*_0_ on the barrier), due to asynchronicity and the related formation energy of the reactant complex.^33^

Third, we identified asynchronicity as a key contributing factor to the rare phenomenon of a reaction rate slowdown with increasing temperature in a single-step HAA process.^34^ This counterintuitive behavior, previously rationalized through strongly temperature-dependent equilibrium constants, can be more comprehensively understood within our framework. By dissecting the barrier into its contributions such as Δ*G*_0_ and asynchronicity, we provide an alternative to the standard Eyring plot analysis, which partitions the barrier into enthalpic and entropic terms.

Fourth, we uncovered that asynchronicity directly modulates the degree of quantum tunneling in HAA (Figure 7).^36^ The tunneling correction to the barrier (Δ*E*_tun_) becomes most pronounced near the synchronous limit (η ≈ 0) and progressively fades as the reaction becomes more ET- or PT-dominated. In other words, reactions in which the proton and electron move in concert exploit tunneling most efficiently, while asynchronous trajectories, where one particle “leads” the other, traverse a broader potential barrier with negligible quantum tunneling assistance. The magnitude of Δ*E*_tun_ typically spans 0 to – 2 kcal mol^–1^, consistent with experimental H/D kinetic isotope effects (H/D KIEs): larger KIEs signify more synchronous and thus more tunneling-favored HAA pathways. In light of recent reports of tunneling contributions in asynchronous (PT-favored) HAA reactions of Co–oxo complexes,^35^ we do not suggest that asynchronous pathways lack tunneling; rather, we propose that tunneling would be even more pronounced in the synchronous limit, provided other factors (e.g., Δ*G*_0_) remain unchanged.

**Figure 7:**
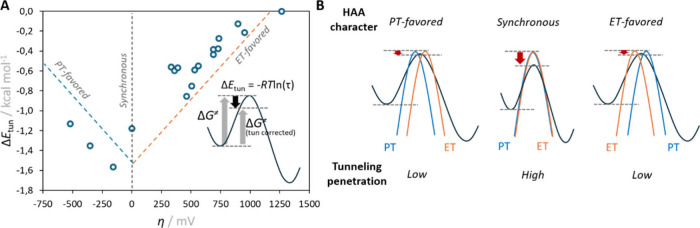
(A) Correlation between asynchronicity η and the Eckart tunneling term Δ*E*_tun_ for reaction systems from Figure 4, which peaks near η ≈ 0 (synchronous HAA) and falls off toward PT- or ET-favored limits. Dashed lines are visual guides; insets relate Δ*E*_tun_ = −*RT* ln­(τ) to the tunneling-corrected barrier. (B) Schematic potential-energy profiles for PT-favored (left), synchronous (center), and ET-favored (right) HAAs, showing how increasing η broadens the barrier and narrows the tunneling window. Adapted with permission from ref 36. Copyright 2019 Royal Society of Chemistry.

Fifth, we found that asynchronicity of a single-step HAA process may affect the product outcome in cases, where HAA products can have more than one (post-HAA) reactive channel available, with low or vanishing kinetic barriers^4^—a scenario where selectivity is dynamically controlled and cannot be rationalized using the statistical transition state theory. In such processes, product distribution is encoded to a large extent in the distribution of atomic momenta at the reactive mode of the transition state of HAA manifested by kinetic energy distribution (KED) within that mode, which is in turn affected by asynchronicity (Figure 8).^36^ Specifically, we showed that synchronous reactions typically feature a motion within TS reactive modes that is more localized on the transferred H atom, leading to a higher fraction of unitless KED on H atom (KED_H_ in Figure 8). This KED localization, in turn, correlates with an increased degree of tunneling τ, which manifests by a higher H/D KIE.

**Figure 8:**
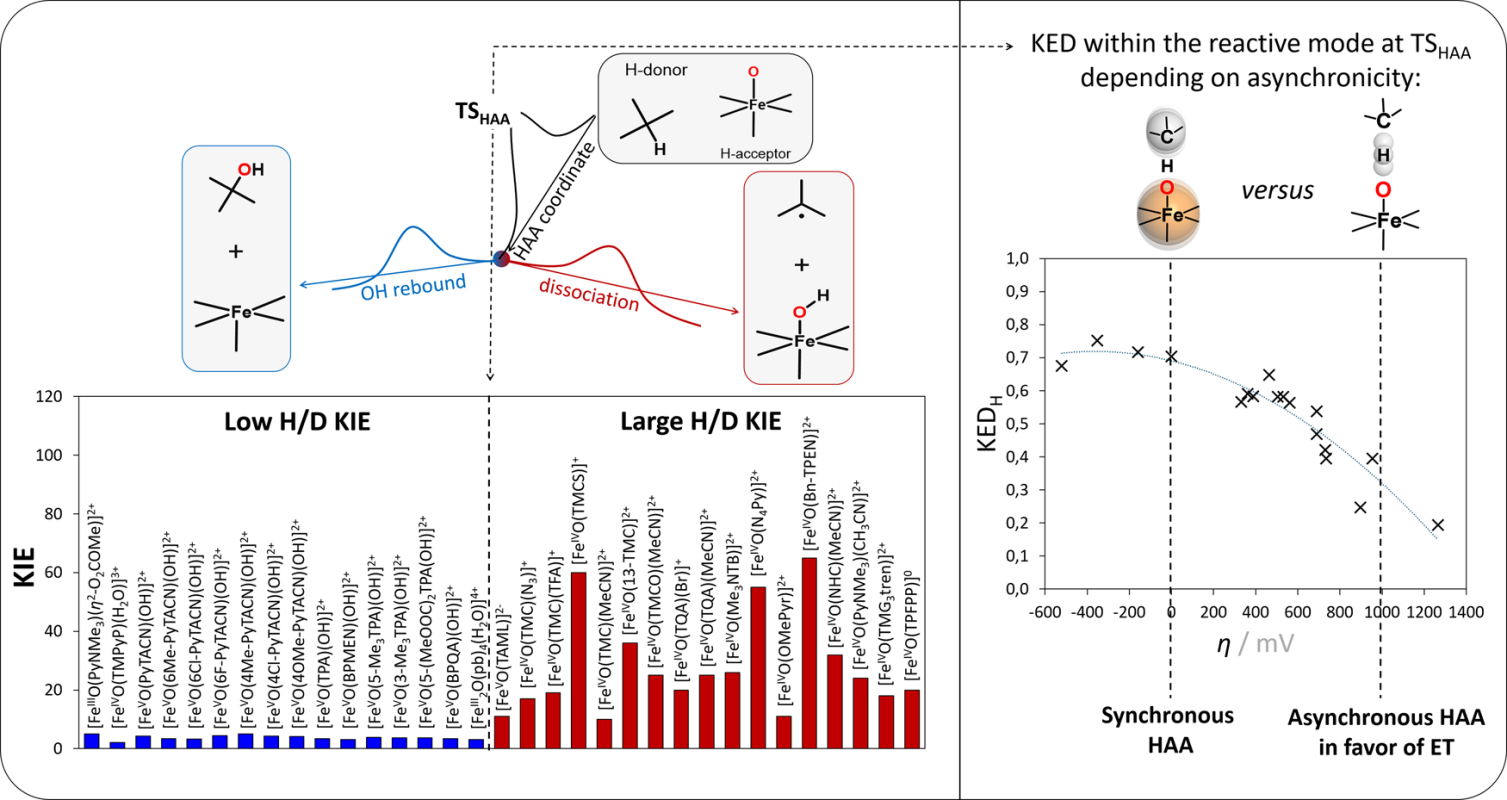
Connection between asynchronicity η and the character of the TS in HAA. Negative η corresponds to H-centered motion (large KED_H_ manifested by a large primary H/D kinetic isotope effect, H/D KIE) associated with PT-driven HAA and post-HAA dissociation, while positive η corresponds to heavy-atom-centered motion (large 1 – KED_H_ manifested by a low H/D KIE) associated with ET-driven HAA and post-HAA OH rebound. Adapted with permission from refs 4 and 36 . Copyrights 2020 and 2019 American Chemical Society and Royal Society of Chemistry, respectively.

The asynchronicity-driven post-HAA selectivity may occur, for example, when a Fe^IV^=O complex abstracts a hydrogen atom from a C–H bond, resulting in the formation of a Fe^III^–OH species and a substrate carbon radical. This radical can either dissociate away from the Fe^III^–OH (radical escape) or proceed via radical rebound mechanism toward hydroxylation, ultimately forming Fe^II^ and a C–OH product. Specifically, we found that reaction systems with a low fraction of kinetic energy concentrated in the transferred H atom at TS_HAA_ (low KED_H_) tend to favor the hydroxylation pathway; contrarily, systems with a high degree of KED_H_ are more likely to undergo radical escape (Figure 8). Considering that the magnitude of KED_H_ influences the extent of tunneling effects and, consequently, H/D KIE, we predicted and observed that systems with low experimental H/D KIE tend to favor hydroxylation, whereas those with high H/D KIE are more prone to radical escape. Thus, our approach offers a low-cost, qualitative yet predictive framework based on asynchronicity (i.e., disparity in redox vs acidobasic thermodynamic driving force) for guiding post-HAA selectivity and highlights the utility of experimental H/D KIEs as mechanistic probes. Notably, the insights from our unconventional analysis were recently supported by an independent molecular dynamics study.^37^

## Frustration as the Second Off-Diagonal Thermodynamic Component: Quantified View of How It Shapes HAA Reactivity

In addition to asynchronicity, we examined the role of the second off-diagonal component—frustration—and its effect on HAA reactivity.^2^ While asynchronicity reflects the imbalance between the proton- and electron-transfer components of HAA, frustration from Figure 3 is best understood in the context of the common inverse relationship between reduction potentials and basicities: strong oxidants are typically weak bases, and vice versa. When a species A^•^ is simultaneously a much stronger oxidant *and* a much stronger base than its reaction partner D^•^ (or, conversely, much weaker in both respects), a conflict arises regarding whether to favor proton or electron transfer in reaction. This conflict manifests as an energetic penalty on the reaction and, therefore, such species are said to be *frustrated*.

To dive in, we have employed two distinct, well-established reaction sets: the first, where Co^III^O acts as the oxidant for 11 different C–H bond substrates (Anderson’s set^19,21^ ), and the second, where Mn^IV^O serves as the oxidant for 10 different C–H bond substrates (Borovik’s set^22^). In this study, we correlated λ_0_ with frustration σ and we found that λ_0_ reaches its minimum when σ approaches to zero (denoted as λ_00_). By incorporating this relationship into the already asynchronicity-enriched, linearized Marcus-type model for the reaction barrier shown in Figure 4, we arrive at the following equation:

2 $$\Delta G_{\mathrm{HAA}}^{⧧}=\frac{\lambda_{00}}{4}+\frac{\Delta G_{0}}{2}+\frac{F}{4}\left(\left|\sigma \right|-\left|\eta \right|\right)$$

The key finding regarding frustration is 2-fold: (i) the free-energy barrier was found to be directly proportional to +|σ|/4, which consistently increases the barrier and (ii) only after incorporating its effect, along with the contributions of Δ*G*_0_ and asynchronicity, the thermodynamic correlation with the HAA barrier yielded quantitatively accurate results for each set (Figure 9). This finding left the remaining term λ_00_/4, a phenomenological term encompassing all factors dependent on the reaction coordinate (e.g., sterics, formation of reactant complex, catalytic participation of additives such as Lewis/Brønsted acids^38–40^ ) Analogous to the *intrinsic* barrier in Marcus theory, this term is expected to be strictly positive. The increase in the energy barrier due to elevated frustration aligns well with the impact of ET and PT on the free-energy surface, as observed in the transition from the left to the middle graph in Figure 2B.

**Figure 9:**
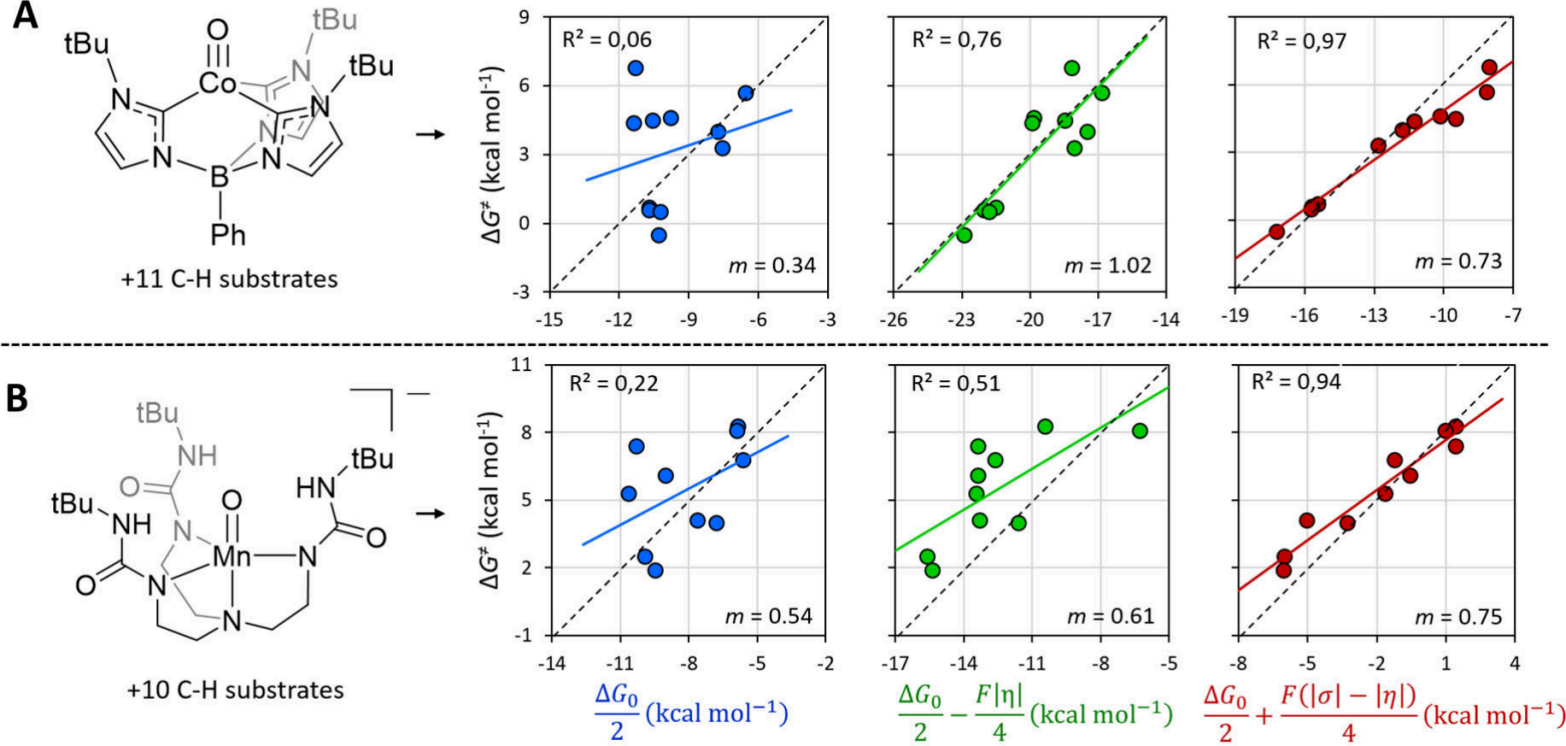
Thermodynamic approximations for predicting HAA barriers compared against Δ*G* ^⧧^ values derived from experimental rate constants. (A) Co^III^–oxo complex with a set of C–H substrates. (B) Mn^IV^–oxo complex with a set of C–H substrates. In each case, three levels of thermodynamic projection are tested: the diagonal contribution (Δ*G*_0_/2), the diagonal plus asynchronicity correction (Δ*G*_0_/2 – *F*|η|/4), and the full three-component thermodynamics (Δ*G*_0_/2 + *F*/4 (|σ| – |η|)). Correlation plots demonstrate the progressive improvement of predictive accuracy across the three formulations. Adapted from ref 2. Copyright 2023 Royal Society of Chemistry.

## Remarks on the Overall Off-Diagonal Thermodynamic Contribution to the Reaction Barrier

From eq 2, the overall off-diagonal thermodynamic contribution to single-step HAA reaction barrier according to the present model is given as

3 $$\Delta G_{\mathrm{off‐diag}}^{⧧}=\frac{F}{4}\left(\left|\sigma \right|-\left|\eta \right|\right)$$

the graphical representation of which is given in Figure 10. The color-coded surface with isocontours represents the contribution of Δ*G*_off‑diag_^⧧^ to the single-step HAA, dependent on both σ and η. Since both σ and η comprise redox and acid–base terms, as discussed earlier in the text, the map also includes two coordinates: *F*Δ*E*° (the difference between the reduction potentials of A^•^ and D^•^) and *RT* ln(10)­Δp*K*_a_, which represents the difference between the acid–base potentials of A^•^ and D^•^. Importantly, Figure 10 highlights several key features associated with the off-diagonal thermodynamic contribution to the reaction barrier. First, the plot can be divided into four distinct zones, where the interplay between frustration and asynchronicity causes Δ*G*_off‑diag_^⧧^ to be dominated by the *RT* ln(10)­Δp*K*_a_ or *F*Δ*E*° terms. This is an important feature, as experimental groups sometimes report correlations between rate constants and the reduction potentials^41,42^ or acidity constants^19,22^ of substrates (reacting with the same oxidant). Second, the isocontours clearly indicate directions along which the off-diagonal term does not contribute to difference in the HAA barrier between reactions. For example, this is the case in ref 43 resulting in a neat LFER. In summary, the presented map may serve as a guide to help chemists design oxidants with tailored selectivity for specific H atom abstraction reactions.

**Figure 10:**
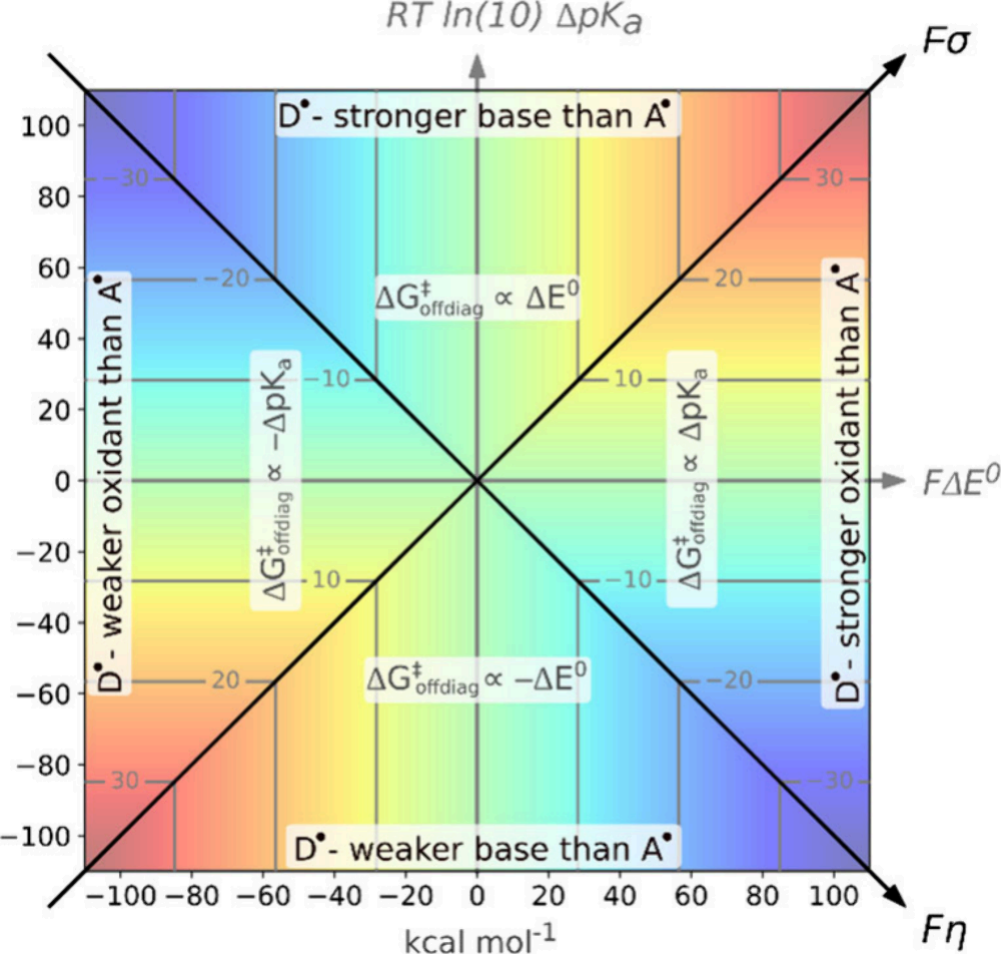
Off-diagonal thermodynamic contribution to the HAA barrier (Δ*G*_off‑diag_^⧧^) as a function of asynchronicity η and frustration σ. The four regions indicate whether Δ*G*_off‑diag_^⧧^ is primarily influenced by a difference in p*K*_a_ between A^•^ and D^•^, or by the difference in their *E*° values. Adapted with permission from ref 2. Copyright 2022 American Chemical Society.

## Extending Three-Component Thermodynamics to Radical Transfer and HAA Mechanisms

So far, we have assumed that proton and electron follow “one direction” from H atom donor to H atom acceptor. We can refer to this situation as a *concerted unidirectional cation/electron transfer*. However, this is not necessarily the case of other radical transfer processes such as a well-known chlorine- or hydroxyl-radical transfer reactions (Cl^•^ or OH^•^ rebound), for example in the nonheme iron halogenase SyrB2, where the Cl^–^ or OH^–^ ligand, coordinated to the Fe^III^ metal center, recombines with the substrate carbon-based radical to produce C–Cl or C–OH products and Fe^II^.^44^ In these cases, the formal radical transfer involves the anion transfer (Cl^–^ or OH^–^) from the radical donor to the radical acceptor, coupled in one single step with a reverse electron transfer from the anion acceptor back to the donor as demonstrated by analysis of diabatic states mixing in the reaction.^44^ Similar mechanism was revealed for a C–F bond activation by intrinsic bonding orbitals.^45^ In analogy to the unidirectional case, we can qualify this scenario as *concerted bidirectional anion/electron transfer*. The thermodynamic cycles and associated half-reactions for these two cases of concerted electron/proton (cation) transfer and concerted electron/anion transfer are presented in Figure 11. Although both cycles share the same diagonal pathway, they differ in their off-diagonal, stepwise pathways. Specifically, the right-hand cycle involves an anion-transfer intermediate (instead of proton/cation transfer) and an electron transfer intermediate, in which the electron is transferred from acceptor to donor (rather than donor to acceptor). These differences in the off-diagonal states reflect a fundamental mechanistic insight into the single-step pathway. We hypothesized and later confirmed through a series of OH rebound reactions involving (difluoro)­cyclohexadienyl radical substrates and model tetramethylcyclam-supported Fe^III^–OH complexes that the reaction mechanism indeed follows the thermodynamic cycle with the more favorable off-diagonal activation free energy Δ*G*_off‑diag_^⧧^, as defined in eq 3.^5^ This mechanistic aspect was demonstrated by the change in density-based charge and volume of the transferred H atom moiety from the reactant to the transition state.

**Figure 11:**
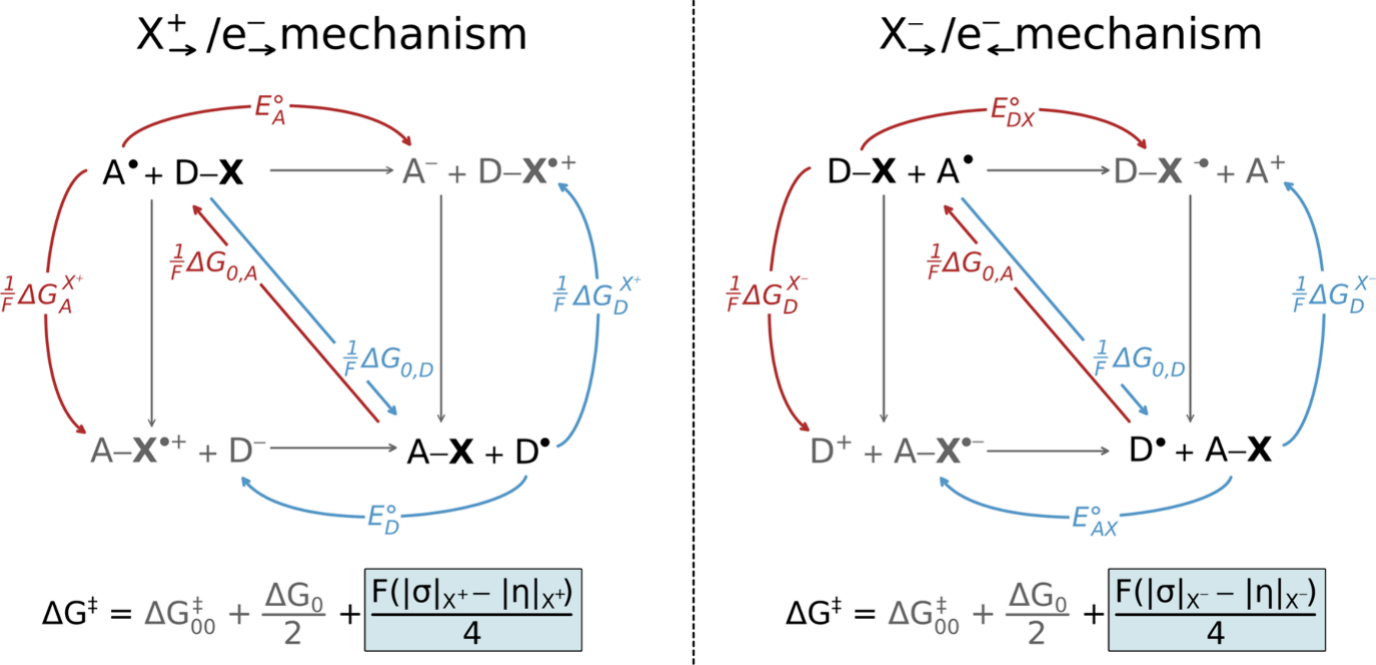
Reaction D–X + A^•^ → D^•^ + X–A with X = OH, Cl, H, ... and associated thermodynamic cycles for cation/electron- and anion/electron-flavored X-radical transfer between donor D–X and acceptor A^•^. Red and blue arrows denote half-reaction blocks defined by (i) *diagonal* X^•^ binding free energies (Δ*G*_0,D_, Δ*G*_0,A_) and (ii) *off-diagonal* terms for X^+^ binding/X^–^ release (Δ*G* ^X^+^^, Δ*G* ^X^–^^) and one-electron reductions of D^•^/A^•^ or D–X/A–X Δ*G*_D_^e^–^^, Δ*G*_A_^e^–^^, Δ*G*_DX_^e^–^^, and Δ*G*_AX_^e^–^^). Off-diagonal quantities yield composite descriptors (potential dualities and disparities) governing asynchronicity and frustration (Figure 3B). These parameters are incorporated into the three-component thermodynamic model describing the X-radical abstraction barrier. Adapted with permission from refs 5 and 6 . Copyrights 2024 and 2025 Royal Society of Chemistry and American Chemical Society, respectively.

Since HAA can be viewed as a specific case within the broader framework of radical transfer chemistry, it is reasonable to expect that an alternative mechanism involving concerted anion/electron transfer could also operate within this chemical space. Thus, by analogy to radical transfer, the two HAA pathways can be depicted as distinct thermodynamic cycles (Figure 11, with X = H), each giving rise to a different value of Δ*G*_off‑diag_^⧧^. One cycle corresponds to the conventional PCET-type HAA, whereas the other describes HAA proceeding via *hydride-coupled electron transfer* (HCET-type HAA). Comparison of the associated Δ*G*_off‑diag_^⧧^ values indicates that HAA between cyclohexane and the experimentally well-characterized Tolman’s Cu^III^–OH complex^46^ proceeds through the HCET-type pathway.^6^

Similar to the previously mentioned OH rebound reaction study, the thermodynamic preference for such mechanism is consistent with the analysis of density-based descriptors—and is further supported by the intrinsic bonding orbital (IBO) analysis shown in Figure 12. In this context, tracking the IBOs of the (α/β) electron pair forming initially the σ bond in the X–H substrate (X = C, O, etc.) within the reactant complex (RC) along the reaction coordinate for PCET-like HAA (shown on the left of Figure 12) reveals that the β electron gradually translocates from the substrate to the metal center’s *d* orbital in the H atom abstractor, while the α-electron remains localized on the substrate radical. In contrast, for HCET-like HAAs (on the right of Figure 12), the electron pair behaves differently: both the α and β electrons from the σ_X–H_ bond gradually move from the substrate to the metal’s *d* orbital as the system progresses from RC to TS. However, the β electron in the post-TS phase returns to the donor, forming the substrate radical. Mechanisms such as PCET-like vs HCET-like HAAs adds to the complexity of HAA chemistry, whereas the detailed understanding is not only of fundamental interest, but it should be carefully considered when predicting reactivity based on thermodynamic properties.

**Figure 12:**
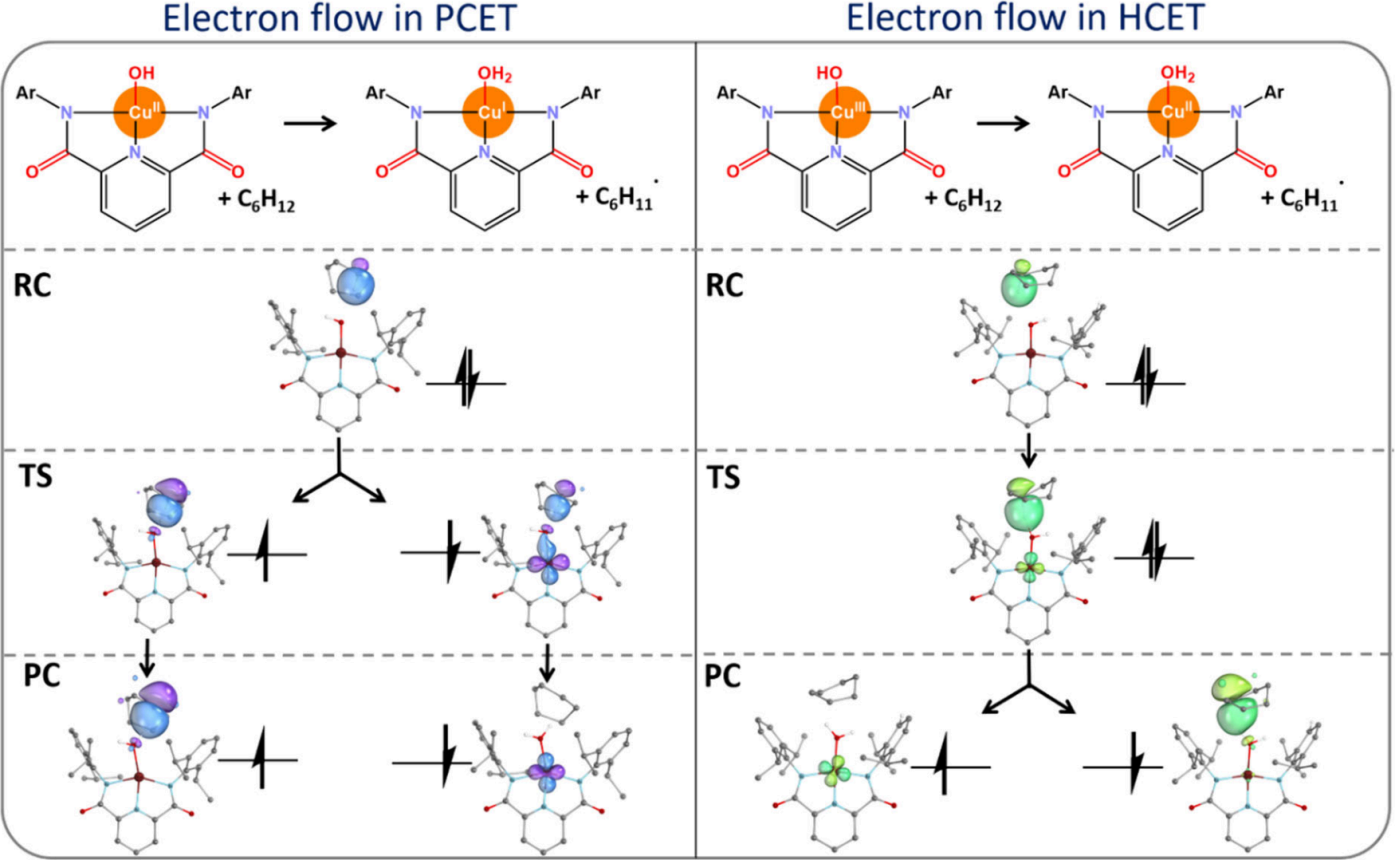
Electron flow in the PCET-like/HCET-like HAA reaction between cyclohexane and the Cu^II^–OH/Cu^III^–OH complex (left/right), observed through intrinsic bond orbital analysis, tracking the fate of the electron pair that initially forms the σ_C–H_ bond in cyclohexane as it progresses through the HAA reaction (from reactant complex to transition state to product complex). Adapted with permission from ref 6. Copyright 2025 American Chemical Society.

## Challenges and Outlooks

Over the past eight years, the thermodynamic principles studied in our group have been supported by others, as noted earlier. However, these principles have mostly been applied a posteriori to analyze and interpret experimental data. Our ultimate goal is to shift this paradigm by using them predictively and intuitively (a priori) for HAA reactivity/selectivity. Nevertheless, the model still has limitations in quantitative predictions of HAA reactivity/selectivity. Technically, one needs to bear in mind that the working model relies predominantly on computational data, and therefore further improvement in its predictive power can benefit from a more systematic validation of the chosen level of quantum-chemical theory. Beyond this, the primary challenges can be summarized as follows:

(1) How can we define *non-empirically* whether the set of HAA reagents and reactions are chemically related? Gaining this insight is expected to provide a better control over the variability in the fourth nonthermodynamic term λ_00_/4 complementing the three-component thermodynamics.
(2) The nonlinear features of the Marcus-type reaction barrier, as expressed in the first equality of eq 1, are not captured by the current linearized model. The omission of this nonlinearity in the second equality of eq 2 leads to the erroneous exclusion of phenomena such as the so-called *inverted* region—a strong non-LFER regime in which the rate decreases with a higher equilibrium constant. Understanding how off-diagonal thermodynamic effects interact with diagonal contributions in the full quadratic model, and how this interplay enhances the model’s predictive accuracy, is an important step toward developing a more quantitative framework.
(3) HAAs encompass reactions ranging from highly nonadiabatic PCET (and potentially HCET) mechanisms, governed by vibronic gating—where structural fluctuations transiently enhance donor–acceptor electronic coupling and proton vibrational wave function overlap, as described by Hammes–Schiffer theory^47^—through moderately adiabatic PCET-like/HCET-like regimes, to strongly adiabatic hydrogen-atom transfers (HATs). The present linearized Marcus-type model for the HAA barrier is well applicable to reactions spanning weakly to strongly adiabatic regimes (some specific examples given in ref 33), in which HAAs proceed on a single potential energy surface with a well-defined TS, while the degree of adiabatic coupling is treated implicitly and absorbed into the λ_00_/4 term and waits for resolution.

To support future developments, we recently expanded the scope of off-diagonal thermodynamics by showing that the Marcus cross-relation (MCR), originally formulated for electron-transfer reactions, can be successfully extended to diverse HAA systems when off-diagonal effects are included.^26^ In MCR, the rate constant between D–H and A^•^ (*k*_DA_) is expressed as the geometric mean of the self-exchange rates (*k*_DD_ and *k*_AA_), adjusted by the reaction equilibrium constant (*K*_DA_), which can also be written in terms of activation free energies and Δ*G*_0_ (Figure 13, top). For HAA, including one-half of Δ*G*_off‑diag_^⧧^ term from eq 3 allows MCR to hold across systems with varying adiabatic couplings—from weakly adiabatic PCET-like HAAs to strongly adiabatic HATs. Overall, MCR provides a foundation for a unified thermodynamic model (beyond eq 2) encompassing both adiabatic and near nonadiabatic HAA, with recent studies aiming to reconcile asynchronicity and nonadiabaticity.^48^

**Figure 13:**
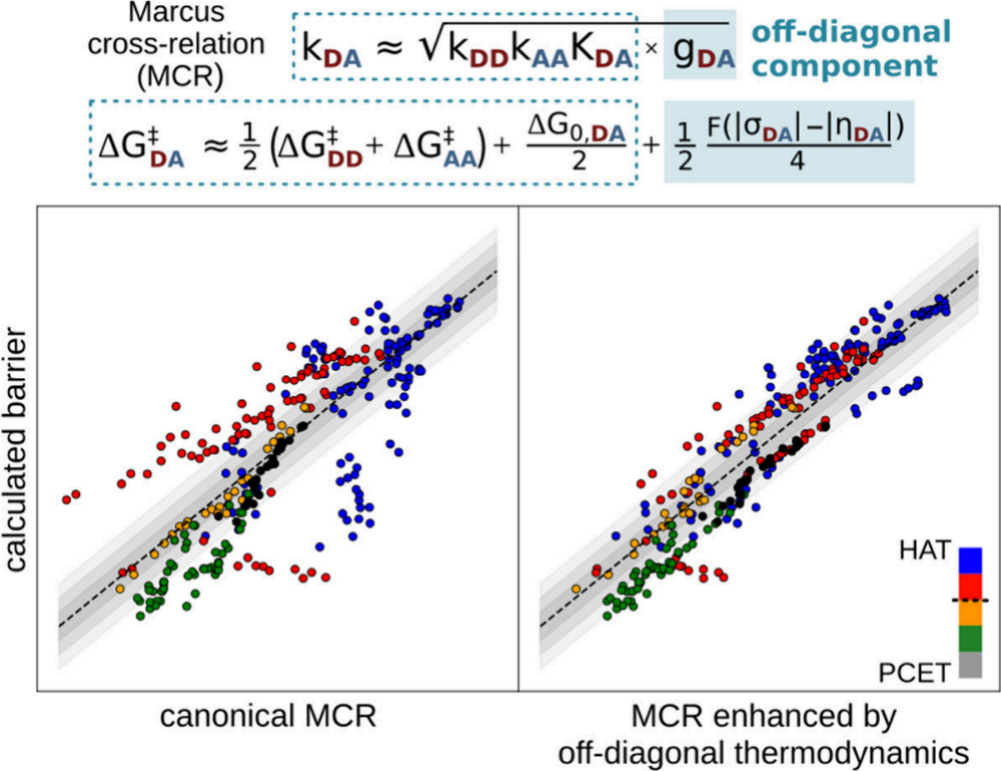
Computed barrier Δ*G*_DA_^^⧧^^ for the HAA reaction between the H atom donor (D) and the acceptor (A) from the left-hand side of the equation (inside the dashed box) compared to the barrier predicted by the standard MCR model from the right side of the equation (inside the dashed box) in the left graph, or Δ*G*_DA_^^⧧^^ vs the MCR barrier corrected by the off-diagonal term (within the blue-filled box) in the right graph. Adapted with permission from ref 33. Copyright 2025 AIP Publishing.

The aim of this Account was to introduce *off-diagonal thermodynamics* and demonstrate its role in hydrogen atom abstraction and related radical transfer reactivity, highlighting its ability to rationalize experimental trends. We showed that *asynchronicity* (typically the dominant off-diagonal component) can direct HAA selectivity in counterintuitive ways, favoring stronger D–H bonds through an asynchronicity-controlled pseudoinverted regime. This framework also quantifies the polarity-match effect in HAA chemistry. Furthermore, off-diagonal thermodynamics—particularly asynchronicity—modulates key HAA features, including adiabatic coupling, tunneling, and post-HAA channel selectivity. It also reveals when and why a distinct pathway, *hydride-coupled electron transfer*, emerges, and extends beyond HAA to other radical-transfer processes. Finally, we outline future steps that are probably necessary to pave the way toward a unified and predictive view of reactivity and selectivity in radical chemistry, offering new avenues for designing catalysts suitable for chemo- and regioselective transformations.
